# Advancements in Precision Prevention: Top Bioinformatics and Translational Informatics Papers of 2023

**DOI:** 10.1055/s-0044-1800724

**Published:** 2025-04-08

**Authors:** Scott McGrath, Mary Lauren Benton

**Affiliations:** 1 CITRIS Health, University of California Berkeley, USA; 2 Department of Computer Science, Baylor University, USA

**Keywords:** Bioinformatics, Genomics, Transcriptomics, Machine learning, Precision medicine

## Abstract

**Objective** : To identify and summarize the top bioinformatics and translational informatics (BTI) papers published in 2023, focusing on the area of precision prevention.

**Methods** : We conducted a literature search to identify the top papers published in 2023 in the field of BTI. Candidate papers from the search were reviewed by the section co-editors and a panel of external reviewers to select the top three papers for this year.

**Results** : Our literature search returned a total of 550 candidate papers, from which we identified our top 10 papers for external review. The papers were evaluated based on their novelty, significance, and quality. After rigorous review, three papers were selected as the top BTI papers for 2023. These papers showcased innovative approaches in leveraging machine learning models, integrating multi-omics data, and developing new experimental techniques. Highlights include advancements in single-cell genomics, dynamic surveillance systems, and multimodal data integration.

**Conclusions** : We found several trends in the ten candidate BTI papers, including the refinement of machine learning models, the expansion of diverse biological datasets, and the development of scalable experimental techniques. These trends reflect the growing importance of bioinformatics and translational informatics as a cornerstone for improving predictive and preventative healthcare measures.

## 1. Introduction

We begin this year's effort to identify the best papers in Bioinformatics and Translational Informatics (BTI) through the lens of digital informatics and in the pursuit of precision in prevention. As the global health landscape evolves, so does our understanding of the multifaceted interactions between technology, data, and human health. This year, we highlight significant scholarly contributions that demonstrated cutting-edge computational techniques to strengthen predictive accuracy, work which helped introduce diverse biomedical data, and efforts that shared novel approaches to enhance precision medicine. The selected papers exemplify the best in BTI and illustrate how digital tools and methodologies can not only anticipate future health challenges but also forge pathways to mitigate them before they manifest. From scalable algorithms capable of deciphering vast genomic landscapes to sophisticated models predicting disease susceptibility and progression, these studies are at the forefront of the shift towards anticipatory health measures.

Noteworthy among this year's highlights are innovations in machine learning models that refine our ability to forecast health outcomes, efforts to synthesize multi-omics data for a comprehensive understanding of disease mechanisms, and the development of dynamic surveillance systems for real-time health monitoring. Each contribution not only pushes the envelope in its respective field but also underscores the transformative potential of digital informatics in fostering advancements from treatment to prevention.

In this review, we share our methodology to identify candidate papers, outline the emerging trends they represent, and discuss their implications for the future of healthcare. Our goal is to help highlight the bioinformatics and translational informatics work that helps move medical science forward, urging the community towards a more informed, precise, and preventive approach to health and well-being.

## 2. Methods

We conducted a literature search using PubMed to identify candidate best papers in bioinformatics and translational informatics published in 2023. We queried PubMed using the following Medical Subject Headings (MeSH) terms and their derivatives: “bioinformatics”, “computational biology”, “translational research, biomedical”, “genetics, medical”, “genomics”, “gene expression”, “transcriptomics”, “proteomics”, “epigenomics”, “metagenomics”, “omics”, “precision medicine”, “algorithms”, and “machine learning”. All results were identified by a combination of at least one of the following computation-related terms (e.g., bioinformatics, machine learning), and at least one of the biology-related terms (e.g., omics). We required all articles to be described by the indexer assigned MeSH term to increase the translational and clinical relevance of the results. Finally, we limited our query to return articles with electronic publication dates between January 1, 2023 and December 31, 2023.

Due to the large number of papers returned by the PubMed query, we prioritized articles published in several key journals for BTI: Journal of the American Medical Informatics Association (JAMIA), Journal of Biomedical Informatics (JBI), PLoS Computational Biology, Bioinformatics, BMC Bioinformatics, BMC Systems Biology, Nature, Nature Genetics, Nature Biotechnology, Nature Methods, Science, Science Translational Medicine, Clinical Pharmacology and Therapeutics, New England Journal of Medicine, Journal of the American Medical Association (JAMA), Lancet, PLoS Genetics, and Cell. Our query resulted in 550 articles for the initial round of editorial review.

In the initial editorial review, we screened the titles and abstracts of all articles to select the ones that were most relevant to BTI. We evaluated each abstract based on three criteria: novelty, significance, and quality. Novelty indicates the originality of the research question or approach; significance indicates the potential impact on the BTI field; and quality indicates the technical, biological, and clinical accuracy of the paper. This process narrowed our selection to 15 articles. During the second round of editorial review, we scored the full text of the articles on the same criteria and ranked them accordingly. This led to a final collection of 10 candidate articles, which were then assessed by external experts and other IMIA editors and scored across six criteria: importance, scientific/practical impact, scientific content, originality, quality of literature, and quality of presentation. From the final round of review, we identified the three top-scoring articles as the best BTI papers of 2023 (see Table 1 ). A summary of the three best papers (and one honorable mention) can be found in the appendix of this synopsis.

**Table 1.** Selection of best papers for the IMIA Yearbook of Medical Informatics for the Bioinformatics and Translational Informatics section. The articles are listed in alphabetical order of the first author's surname. The * denotes the honorable mention.

Section Bioinformatics and Translational Informatics
Bhattacharya A, Vo DD, Jops C, et al. Isoform-level transcriptome-wide association uncovers genetic risk mechanisms for neuropsychiatric disorders in the human brain. Nat Genet. 2023 Dec;55(12):2117-2128. doi: 10.1038/s41588-023-01560-2. Li Y, Guo Z, Gao X, Wang G. MMCL-CDR: enhancing cancer drug response prediction with multi-omics and morphology images contrastive representation learning. Bioinformatics. 2023 Dec 1;39(12):btad734. doi: 10.1093/bioinformatics/btad734. Theodoris CV, Xiao L, Chopra A, et al. Transfer learning enables predictions in network biology. Nature. 2023 Jun;618(7965):616-624. doi: 10.1038/s41586-023-06139-9. *Liao WW, Asri M, Ebler J, et al. A draft human pangenome reference. Nature. 2023 May;617(7960):312-324. doi: 10.1038/s41586-023-05896-x.

## 3. Themes

The ten candidate best papers for 2023 illustrate recent efforts towards enhancing predictive models of disease by integrating large-scale biological data with novel machine learning techniques. Below, we briefly discuss several main themes identified while evaluating the top BTI papers and highlight their connections with precision medicine and prevention.

### 3.1. Enhancing Single-cell Genomics and Data Generation for Novel Insights

Current research in BTI continues to leverage the rapid improvements in -omics profiling and integration of biomedical imaging to answer complex questions about disease biology. Many of these datasets and experimental techniques are well-established in the field; however, the candidate best papers from 2023 highlight novel extensions and applications.

For example, Fonseca et al. developed a new single-cell atlas for a clinically relevant, but understudied, tissue type [ 1 ]. They conducted a single-cell transcriptomic study of endometrial tissues with the goal of improving our understanding of endometriosis. In diseases such as endometriosis, where profiling bulk tissues is likely to miss molecular hallmarks of disease and disease subtypes, these atlases hold great promise for new precision treatments. Johnson et al. [ 2 ] developed an atlas detailing the enzyme specificity of more than 300 serine/threonine kinases, annotating the relationships between kinases and their substrate motifs. Since previous work has linked thousands of phosphorylation sites to disease processes, this atlas can allow future studies to better predict the changes to signaling pathways and cellular processes that result from perturbations to the kinome. In Walker et al. [ 3 ], researchers studying the molecular and genetic underpinnings of Type II diabetes (T2D) generated large, multi-modal datasets to integrate features across pancreatic islets from cases and matched controls. They found that genome-wide association study (GWAS) variants associated with T2D were enriched in co-expression networks associated with physiological features, including the network containing RF6X. This paper highlights how integrating data across modalities and cell types has the power to improve our understanding of molecular mechanisms of disease, and to connect features across scales.

Advances in data generation also increase the translational potential of current research. For example, Clark et al. [ 4 ] presented particle-templated instant partition sequencing (PIP-seq), a novel experimental technique designed to improve the scalability and accessibility of single-cell RNA-sequencing (scRNA-seq). The approach improved on the state-of-the-art and performed well in case studies of clinical samples. For example, in a study of mixed-phenotype acute leukemia, PIP-seq identified transcriptional signatures associated with disease progression and captured the tumor heterogeneity better than traditional approaches. Ultimately, high-throughput and user-friendly approaches such as PIP-seq will increase the accessibility of these techniques in the clinic and open new doors to precision medicine in the form of scRNA-seq monitoring of patient samples. In another study, Morris et al. [ 5 ] developed Systematic Targeting Inhibition of Noncoding GWAS loci with single-cell sequencing (STING-seq), an approach that applies massively parallel CRISPR screens to understand the effects of GWAS variants on blood phenotypes. With the continued investment in large, multi-ancestry biobanks, thousands of genetic variants have been associated with disease; however, even in 2023, it remained challenging to link genetic variants in non-protein-coding regions of the genome to their target genes. These variants are hypothesized to be involved in gene-regulatory functions, but it is time-consuming to experimentally validate these loci. Using STING-seq, the authors tested variants in 254 loci with external evidence of regulatory function. They found target genes for 36% of the tested loci, many of which were not identifiable with previous data. Future work using this approach could connect an even greater number of GWAS loci to disease mechanisms through their involvement in transcriptional regulation.

### 3.2. Maximizing performance with transfer learning and data integration

Alongside continued improvements in data generation, this year's candidate papers include several that explore the use of new statistical and machine learning techniques to maximize the impact of existing datasets. For example, the use of transfer learning in computational biology has the power to offer substantial insights in applications with limited data, such as disease modeling with scRNA-seq.

One example of transfer learning, Geneformer [ 6 ], is a context-aware, attention-based deep learning model. Geneformer is pre-trained on a corpus of millions of single-cell transcriptomes and can be fine-tuned for applications such as predicting dosage-sensitive genes, identifying therapeutic targets for cardiomyopathy, and distinguishing cell types in diverse tissues like the heart and liver. Similarly, De Donno et al. [ 7 ] developed scPoli (single-cell population level integration), a generative method that learns cell and sample representations to facilitate integration and meta-analysis of single-cell data. In addition to outperforming existing models, scPoli was scalable and able to transfer labels between datasets without access to the full reference dataset. This is a benefit for datasets where the underlying reference data cannot be shared due to computational limitations or privacy concerns. Ranjbari and Arslanturk [ 8 ] described a new method that couples a knowledge distillation framework with a variational autoencoder to predict disease progression in breast and kidney cancer patients. One of the challenges of multi-modal data integration is the large number of heterogeneous features; the knowledge-distillation step of this model helped to concisely and accurately represent relevant features. Bhattacharya et al. [ 9 ], presented the Isoform-level Transcriptome-Wide Association Study (isoTWAS), a technique to impute isoform expression and associate the imputed isoforms with phenotype. The isoTWAS increased power and accuracy for these association tests, particularly in regions with high levels of alternative splicing (such as brain tissues). Using isoTWAS to detect associations with neuropsychiatric traits significantly outperformed gene-level models and provided specific insight into the transcriptional patterns underlying genetic associations. Ultimately, continued development of these (and similar) models has the potential to maximize the usefulness of biological models, particularly in the presence of data heterogeneity.

### 3.3. Applying New Machine Learning Paradigms to ological and Clinical Data

This year, researchers continued to adapt new machine learning techniques to biological datasets and improve performance in translationally relevant models. Li et al. [ 10 ] developed MMCL-CDR (Multimodal Contrastive Learning for Cancer Drug Responses), a machine learning model for predicting drug resistance and sensitivity in cancer cell lines. This model uses deep learning architecture paired with contrastive learning to generate a cellular representation for cancer cell lines from multi-modal data, including gene expression, genetic variation, and histology images. This is paired with a graph convolutional network used to generate vector representations of drugs based on their chemical structures. These two representations are used as input to a multilayer perceptron to predict the resistance/sensitivity of a given cell line to a drug. This model architecture is new in the field of cancer genomics, extensible to other types of -omics data that could improve performance, and has the potential to inform new cancer treatments.

Additionally, Jeong et al. [ 11 ] proposed the Gene-level biomarker discovery from multi-Omics data using graph ATtention neural network (GOAT), that leverages gene-gene interaction networks to identify disease biomarkers. Although incorporating biological networks is common in bioinformatics analyses, GOAT uses a graph neural network (GNN) with attention weights to isolate candidate biomarkers and is able to model complex multi-omics data. Gainza et al. [ 12 ] also applied a novel deep learning method to biological networks. In their study of protein-protein interactions the authors build on Molecular Surface Interaction Fingerprinting (MaSIF), a geometric deep learning model, to develop a framework that can be used to identify protein sites and their complementary binding seeds in silico. Although still in the early stages of development, the ability to design protein binders has the potential to greatly improve drug design.

### 3.4. Boosting future research with multiscale data analysis

Our last BTI theme for 2023 involves exploring biological data across multiple scales to improve our understanding of diseases processes and predictions. On the longest timescale, incorporating data across species helps us better understand the evolutionary events and processes that can influence current disease risk. For example, Keough et al. [ 13 ] compiled data from 241 mammals to identify sequences that have undergone accelerated evolution in humans. Here, the authors showed that many of the human accelerated regions identified also function as neurodevelopmental gene-regulatory enhancers and are associated with species-specific genetic variation. These sequences could provide new insights into human-specific traits and contribute to future efforts to model functional genomics. Generating new tools for real-time modeling of biological data can also positively contribute to human health. Gangavarapu et al. [ 14 ] developed outbreak.info, a scalable and user-friendly website that tracks SARS-CoV-2 evolution. The analysis tools and visualizations provide both researchers and the public with a way to track the SARS-CoV-2 variants and variant locations in close to real-time. Increased accessibility of large amounts of genomic data can improve virus surveillance efforts and help target preventative public health measures.

Finally, the work by Liao et al. [ 15 ] highlights the importance of developing new ways to represent the diversity of human genomes for future research. In this paper, the Human Pangenome Reference Consortium published the first draft of a human pangenome reference. Derived from 47 different individuals, the human pangenome is a new way to represent genetic data that can capture and incorporate individual variability. Analyses using the draft pangenome show substantial improvements over the traditional human reference sequence, including fewer errors in small variant discovery and increased detection of larger structural variants. Realizing the dream of precision medicine will require the use of advanced data structures such as the human pangenome to incorporate the breadth of human genetic diversity into bioinformatics and translational informatics research.

## 4. Conclusion

Our review of the top BTI papers from 2023 underscores advancements in the field, particularly around precision prevention. These studies collectively illustrate the potential of integrating cutting-edge computational techniques with comprehensive biological data to predict and mitigate future health challenges.

Key trends identified in this year's top papers include the refinement of machine learning models to enhance predictive accuracy, the integration of multi-omics data for a more holistic approach to understanding of disease mechanisms, and the development of innovative experimental techniques to improve data generation and scalability. For instance, advances in single-cell genomics and the creation of dynamic surveillance systems highlight the growing capability to monitor and predict disease progression in real time. Moreover, the application of novel machine learning paradigms, such as transfer learning and multimodal contrastive learning, has shown promising results. These approaches not only enhance the precision of disease predictions but also open new avenues for personalized therapeutic strategies.

The insights gained from these studies emphasize the critical role of digital informatics in advancing precision medicine. By leveraging diverse and extensive datasets, researchers can develop more accurate models and methods that address the complexities of human health. The importance of a growing diverse dataset to better reflect the global population underscores the importance of the honorable mention this year, the human pangenome. The continued investment in and development of these technologies is essential for realizing the full potential of precision prevention, ultimately leading to better health outcomes and more effective interventions. The integration of sophisticated computational tools and comprehensive biological data continues to push the boundaries of what is possible in precision medicine. Given the rapid advance of artificial intelligence over the past 12 to 14 months, we are eager to see what compelling and exciting work emerges in 2024 for our next annual search for the top BTI papers.
